# Efficacy and Safety of Intravenous Ferric Carboxymaltose Treatment of Iron Deficiency Anaemia in Patients with Corpus Atrophic Gastritis: A Retrospective Study

**DOI:** 10.3390/nu15194199

**Published:** 2023-09-28

**Authors:** Ludovica Dottori, Daniil Corleone Tsar'kov, Emanuele Dilaghi, Giulia Pivetta, Silvia Scalamonti, Irene Ligato, Gianluca Esposito, Bruno Annibale, Edith Lahner

**Affiliations:** Department of Medical-Surgical Sciences and Translational Medicine, Sant’Andrea Hospital, Sapienza University of Rome, 00189 Rome, Italygianluca.esposito@uniroma1.it (G.E.); bruno.annibale@uniroma1.it (B.A.)

**Keywords:** atrophic gastritis, iron malabsorption, iron deficiency anaemia, ferric carboxymaltose, intravenous iron therapy

## Abstract

Corpus Atrophic Gastritis (CAG) is characterised by iron malabsorption leading to iron deficiency anaemia (IDA), which rarely responds to oral therapy. Ferric carboxymaltose (FCM), shown to be a safe and effective intravenous iron therapy in other diseases, has not been investigated yet in CAG. Thus, we aimed to assess the safety and efficacy of FCM in CAG-related IDA. A retrospective study on 91 patients identified CAG as the only cause of IDA treated with FCM. Twenty-three were excluded for incomplete follow-up. Sixty-eight were evaluated for safety and efficacy, while three were evaluated for safety only due to infusion interruption for side effects. Haemoglobin and iron storage were evaluated pre-infusion (T0), at 4 weeks (T4) and 12 weeks (T12) after infusion. An eventual IDA relapse was analysed. Two cases reported mild side effects. Haemoglobin significantly increased at T4, and T12, reaching +3.1 g/dL. Ferritin increased at T4, decreasing at T12, while transferrin saturation increased progressively until reaching a plateau. IDA relapsed in 55.4% of patients at a mean of 24.6 months. The only factor associated with relapse was female gender [OR (95% CI): 6.6 (1.5–28.6)]. FCM proved to be safe and effective in treating CAG-related IDA, ensuring quick and long-lasting recovery.

## 1. Introduction

Corpus atrophic gastritis (CAG) is a chronic inflammatory condition characterised by the destruction of gastric oxyntic mucosa and its replacement by pseudopyloric metaplasia (PPM), intestinal metaplasia (IM), or fibrosis [1]. The consequent loss of oxyntic glands, which are responsible for hydrochloric acid and intrinsic factor release, respectively, essential for iron and cobalamin absorption, leads to possible iron and cobalamin deficiency and, over time, anaemia [2,3].

Anaemia is, in fact, one of the hallmark manifestations of CAG: pernicious anaemia (PA), the megaloblastic macrocytic anaemia due to cobalamin deficiency, is considered a late-stage clinical manifestation [4], while iron deficiency anaemia (IDA) has been shown to arise earlier in CAG natural history, mainly in young women, affecting about 50% of patients [1,2,5].

IDA is a major cause of morbidity and burden worldwide and has relevant consequences on quality of life, cognitive and physical performance, and pregnancy; it is associated with increased mortality and hospitalisation in patients with chronic heart failure and may be a precipitating factor for ischemic cardiac disease [6,7]. Thus, effective and timely treatment of IDA is clinically relevant. Oral iron therapy has some pitfalls and may not be the best therapy option for patients with CAG, at least for two reasons: (i) oral iron therapy is burdened by many side effects, reported up to 70% and mainly involving the gastrointestinal tract [8]; (ii) CAG has been shown to be among the principal causes of oral iron therapy refractoriness. Annibale et al. [9] found that 27% of 71 patients with IDA refractory to oral iron therapy had CAG, while in a study on 150 patients by Hershko et al. [10], the rate of refractoriness to oral iron therapy of IDA patients with hypergastrinemia and anti-parietal cell antibodies or *H. pylori* (Hp) positivity, both highly suggestive of CAG, was about 70%.

Intravenous iron infusion may represent a valid therapeutic alternative in cases of intolerance, ineffectiveness, or contraindication of oral iron therapy [11]. In the past few years, many intravenous iron formulations have been developed, all combined with a carbohydrate to stabilise the complex and reduce hypersensitivity [12].

Ferric carboxymaltose (FCM) is one of the newest preparations and allows administering intravenously higher single doses in a short period of time [13,14]. FCM is made to guarantee a controlled release from the reticuloendothelial system to transferrin, avoiding potentially relevant amounts of ionic iron being released in plasma. FCM has been approved to be administered intravenously in a single high dose, with a maximum of 1000 mg in Europe and 750 mg in the USA, in a short time (15 min) [15,16]. Since its release, FCM has been studied for the treatment of IDA related to gastrointestinal conditions other than CAG, mainly inflammatory bowel diseases (IBD), showing efficacy and tolerability both in clinical trials and post-marketing phase studies [17,18,19,20,21,22].

To the best of our knowledge, FCM use in treating CAG-related IDA has not been investigated so far, neither by clinically randomised trials nor by real-life studies. Thus, this study aimed to assess the safety and efficacy of intravenous FCM in treating IDA due to CAG.

## 2. Materials and Methods

### 2.1. Study Population and Study Design

The current study was designed as a single-centre, retrospective study involving 91 patients with previous CAG diagnoses followed in a teaching hospital, a referral centre for gastric autoimmunity, and atrophic gastritis who received intravenous FCM treatment from March 2017 to July 2021.

These patients were followed-up with an annual medical examination with both clinical and biochemical (complete blood count, including Red Blood Cells Distribution Width (RDW), ferritin, transferrin, iron levels, transferrin saturation, and cobalamin levels) check-ups and with an endoscopic follow-up every 3 years, according to MAPS-II guidelines on precancerous gastric lesions, in order to promptly detect early gastric carcinomas or type 1 gastric neuroendocrine tumours [23,24,25]. The soluble transferrin receptor was not assessed because its dosage is not available in our laboratories.

Patients with Hp infection were cured with a therapy scheme not including proton pump inhibitors due to the corpus atrophy and the consequent hypochloridria present in these patients.

Clinical, serological, endoscopic, and histological data of each patient were accurately registered in an appropriate database, which was used for patients’ management and data analyses.

Inclusion criteria were as follows: adult (>18 years) patients with a diagnosis of CAG, histologically confirmed on gastric biopsies according to the updated Sydney system [26], and with the presence of IDA, as defined below, irrespective of cobalamin deficiency, who underwent intravenous FCM treatment. Patients were excluded when causes of IDA other than CAG, such as overt bleeding, cardiological comorbidities, pneumatological, nephrological, gynaecological, or neurological causes, coagulation diseases, and/or trauma, were present. All patients with IDA aged over 45 years underwent complete colonoscopy; those aged less than 45 years underwent colonoscopy when they had a first-degree relative for colorectal cancer or at least one positive faecal occult blood test assessed on three samples in those with negative family history. For women, an accurate evaluation of menstrual bleeding was conducted using a validated pictorial bleeding chart (Higham score, normal <160). Women with increased menstrual losses were excluded from the study. Patients were also excluded when haematological or clinical follow-up was incomplete.

As discussed later as a limitation of the study, the exact menopausal status was recorded whenever possible and not for all female patients.

The endpoints of the study were: (i) to assess the safety and tolerability of intravenous FCM (endpoint 1: “safety”), and (ii) to assess the efficacy of intravenous FCM treatment in terms of recovery from IDA and in terms of timing for retreatment need, defined as “relapse” (endpoint 2: “efficacy”).

Written informed consent was provided by all participants, and approval of the local ethical committee was achieved (No. CE 7004_2020).

**Figure 1:**
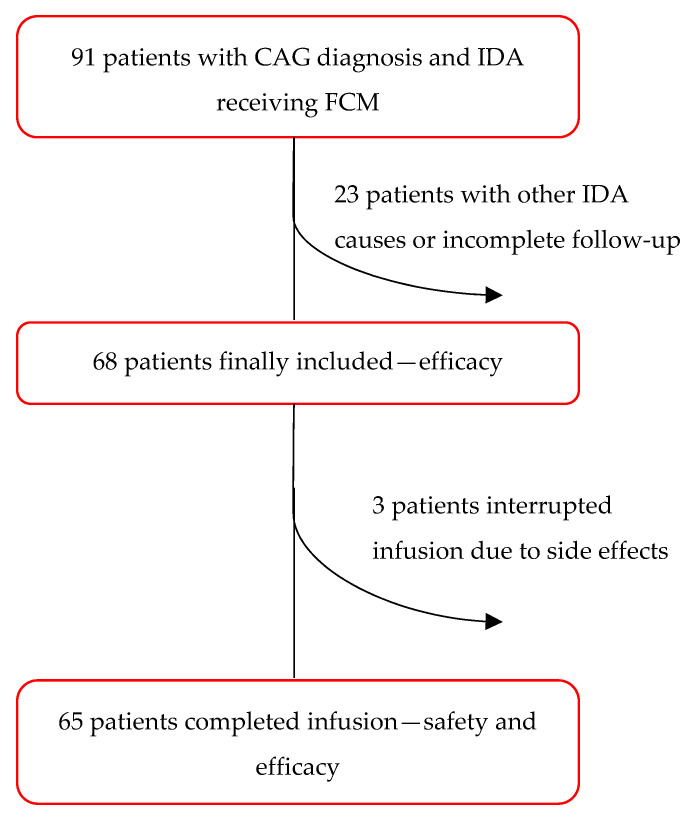
Flow chart.

According to the above-reported criteria, 68 patients (F 78%, median age 61; range 18–92) were finally included in the study (Figure 1). As shown in Figure 1, of the 68 patients, only 65 completed the infusion.

### 2.2. Intravenous FCM Administration and Biochemical and Clinical Follow-Up

Complete blood count, ferritin, transferrin, and iron blood levels were evaluated at the time of intravenous infusion (T0), at 4 (T4), and 12 weeks (T12) after intravenous infusion. FCM dosage was based on haemoglobin (Hb) levels at T0, following the FCM data sheet, and calculated as follows: Hb < 10 g/dL: FCM 1000 mg; Hb > 10 g/dL: FCM 700 mg. No repeated infusions were administered, irrespective of Hb or ferritin levels.

The FCM preparation was diluted in 250 cc sodium chloride 9 mg/ mL (0.9%) sterile solution for intravenous infusion and administered in 30 min. During and after the intravenous infusion, patients were accurately observed by a specialised nurse for half an hour, and any adverse events were promptly evaluated for the eventual need for interruption or medical therapy.

### 2.3. Definitions

CAG was defined as the presence of corpus atrophy, with substitution of oxyntic mucosa by IM or PPM, irrespective of antrum involvement. CAG was histologically diagnosed with esophagogastroduodenoscopy with biopsies according to the updated Sydney system (2 biopsies from the corpus, 2 biopsies from the antrum, and 1 biopsy from the incisura-angularis) [25,26]. Histopathological assessment was performed by an expert pathologist (EP). The histopathological report was redacted according to the criteria of the updated Sydney system [25,26].

The presence of corpus atrophy was graded on a four-grade scale: absence of replacement (score 0), replacement to a mild degree (score 1), moderate degree (score 2), or severe degree (score 3).

IDA was defined as Hb < 13 g/dL in men and <12 g/dL in women, according to the WHO [27], associated with ferritin serum levels <45 ng/mL, according to AGA guidelines [28]. Transferrin saturation was calculated as the ratio between serum iron and the total iron-binding capacity of the available transferrin; a cut-off of 20% was used to define iron deficiency [29]. Anaemia was defined as severe when Hb was less than 8 g/dL, moderate when between 8 g/dL and 10 g/dL, and mild when more than 10 g/dL.

The response to intravenous FCM treatment was defined as normalization of Hb levels (Hb > 13 g/dL for men and Hb > 12 g/dL for women).

IDA relapse was defined as the recurrence of serum Hb levels <13 g/dL for men and <12 g/dL for women.

Adverse events were defined as mild, moderate, and severe according to the severity of manifestations and intervention needed.

### 2.4. Statistical Analysis

Data from patients were expressed as numbers (%) and mean ± standard deviation (SD). A Student’s *t*-test was used to determine differences between haematological values at T0, T4, and T12. A multivariate analysis with logistic regression was conducted to analyse factors possibly associated with relapse (NSAIDs, gender, age > 55 years, concomitant cobalamin deficiency, severe anaemia).

## 3. Results

### 3.1. Study Population

According to the above-reported criteria, 68 patients (F 78%, median age 61; range 18–92) were finally included in the study (Figure 1). In 14 patients (20.6%), previous oral iron therapy was used without success. In 42 patients (61.8%), a concomitant cobalamin deficiency was present. Severe atrophy was present in 25 patients (36.8%), while 58 patients (85.3%) had corpus-restricted atrophy. Six patients (8.8%) had *Helicobacter pylori* (Hp) infection at baseline and were cured. Anti-parietal cell antibodies were positive in 46 of 62 patients (74.1%). 

### 3.2. Timing and Dosage of Intravenous FCM Treatment 

The mean time from CAG diagnosis to intravenous FCM treatment was 20.1 ± 3.5 (months ± SEM), with 62.6% of patients receiving FCM within 1 year of diagnosis.

The FCM dosage available for 55 patients was 1000 mg in 58.6% of patients and 700 mg in 41.4% of patients based on pre-treatment Hb levels or 10 g/dL. None of the patients required additional infusions between T4 and T12.

### 3.3. Safety

Of the sixty-eight patients included in the analysis for safety, three patients (4.4%) reported mild side effects (nausea and dizziness), which, notwithstanding their mild intensity, led to infusion interruption upon patients’ request. These three cases had short-term symptoms (<30 min) and did not require further treatment or hospitalisation.

Among the other sixty-five patients (95.6%) who completed the FCM infusion, just two patients reported very mild side effects (nausea, flushing, and skin eruption) not requiring treatment interruption. These patients were treated with intravenous hydrocortisone (200 mg up to 500 mg) and chlorpheniramine maleate (5 mg up to 10 mg) and completed the FCM infusion. None of the patients reported moderate or severe adverse events or deaths. In the following weeks, no late adverse effects of FCM administration were reported.

### 3.4. Efficacy

#### 3.4.1. Anaemia and Iron Storage Recovery

The 65 patients who received a full FCM dose were evaluated for efficacy; of these, 78.5% were female (*n* = 51), mean age ± SD 58.4 ± 18.3. At baseline (T0), mean Hb was 10.2 ± 1.7 (g/dL ± SD), mean ferritin was 13 ± 29.3 (ng/mL ± SD), and the mean transferrin saturation was 6.1 ± 4 (% ± SD). At 4 weeks (T4), the Hb mean level was 12.6 ± 1.4 (g/dL ± SD), the mean ferritin was 108 ± 106 (ng/mL ± SD), and the mean transferrin saturation was 21.2 ± 7.8 (% ± SD); respectively, it increased with a significance level (*p*-value ≤ 0.0001) of 2 g/dL, >100 ng/mL, and 15%. At 12 (T12) weeks, the Hb mean level was 13.3 ± 1.3 (g/dL ± SD), the mean ferritin was 46.95 ± 75 (ng/mL ± SD), and the mean transferrin saturation was 18.7 ± 9.9 (% ± SD), with further increases in Hb but a decrease in ferritin and transferrin saturation. The Hb levels trend showed an initial rapid increase at T4, followed by a moderated increase at T12, reaching a global increase of 3 g/dL from T0 to T12. Overall, 40 patients reached an increase in Hb levels ≥ 2 g/dL.

Ferritin levels and transferrin saturation levels showed an initial increase at T4, followed by a decrease: ferritin considerably decreased until nearly half of the level reached at T4, while transferrin saturation slightly decreased with respect to T12, substantially describing a plateau.

RDW had no significant increase at T4, significantly decreasing nearly to the initial levels at T12.

**Figure 2:**
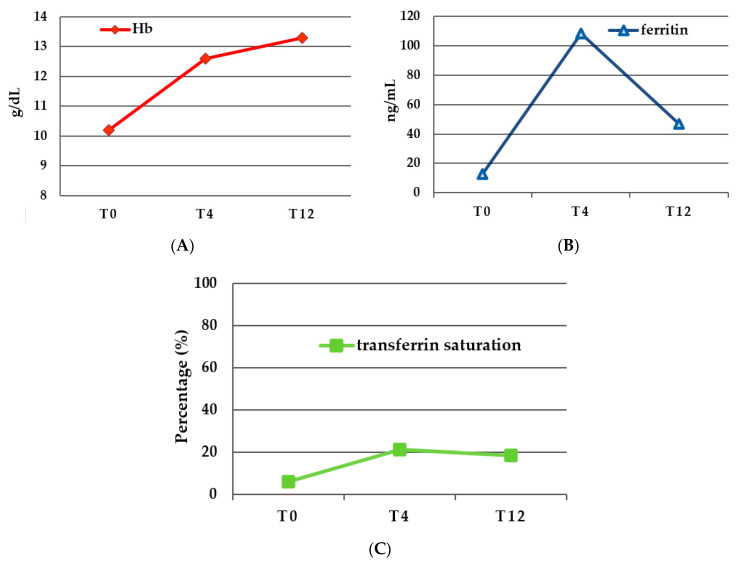
(**A**) Haemoglobin levels (g/dL) at baseline (T0) and at 4 weeks (T4) and 12 weeks (T12) after treatment; (**B**) ferritin levels (ng/mL) at baseline (T0) and at 4 weeks (T4) and 12 weeks (T12) after treatment; and (**C**) transferrin saturation level (%) at baseline (T0) and at 4 weeks (T4) and 12 weeks (T12) after treatment.

All, Hb, ferritin, transferrin saturation, and RDW values at T0, T4, T12, and significance levels of variations are reported in Table 1 and Figure 2.

Recovery from IDA was achieved in 67.3% of patients at 4 weeks and 78.4% of patients at 12 weeks, respectively.

#### 3.4.2. Relapse and Long-Term Follow-Up

Relapse of IDA and need for retreatment occurred in 55.4% of patients after a mean of 24.6 ± 19 (months ± SD). Mean Hb at relapse was 10.5 ± 1.2 (g/dL ± SD), superimposable to the T0 value.

Relapse was caused by CAG only in 95.5% of cases, gynaecological bleeding in 3%, and nephrological causes in 1.5% of cases. 

Patients who relapsed had a mean age of 56.5 ± 17.9 (age ± SD) and 50.8% were female.

In logistic regression, factors associated with relapse were evaluated: gender, age, NSAID use, cobalamin supplementation, severe atrophy, and moderate-severe anaemia. Female gender only was associated with relapse, OR 8.7 [95% CI: 1.4–55.6].

At 1 year after treatment, 57% of patients were free from anaemia. At 2 years after treatment, 38.5% of patients remained free from anaemia.

Characteristics of patients who relapsed and patients who did not relapse are shown in Table 2. Patients who relapsed were more frequently female (*p* = 0.0042); age >55 years, cobalamin supplementation, and severe atrophy were more frequent in relapse (*p* > 0.05); NSAID use was more frequent in non-relapsers (*p* > 0.05); and severe anaemia was equally frequent in both groups (*p* > 0.05).

## 4. Discussion

IDA is one of the most common clinical manifestations of CAG, often leading to its diagnosis. Typically, IDA arises at an earlier stage compared to pernicious anaemia, which is more characteristic of the later stage of the disease [2]. In a study on 160 anaemic patients, IDA was the presenting manifestation of CAG in 52% of patients [9], while in autoimmune atrophic gastritis, IDA was found in about 50% of patients [5]. Given its high prevalence, iron storage restoration is clinically relevant in the management of CAG patients. Oral iron therapy seems to be poorly effective due to the intrinsic impairment of iron absorption in CAG. Of the 71 patients with IDA refractory to oral iron therapy, CAG was found to be the cause of refractoriness in 27% [9], while in a study by Hershko et al. [10], about 70% of patients with hypergastrinemia and anti-parietal cells antibodies or Hp infection, highly suggestive of CAG, had refractoriness to oral iron therapy. Moreover, oral iron therapy is poorly accepted by patients due to annoying, mainly gastrointestinal side effects, which are reported in nearly 70% of patients [8].

Thus, intravenous iron therapy seems to be an eligible strategy for the treatment of IDA in CAG patients. FCM is an intravenous preparation approved in more than 70 countries all over the world for IDA treatment and found to be effective in numerous randomised trials, mainly in patients with chronic heart failure, chronic kidney disease, and IBD [30,31,32].

To the best of our knowledge, this is the first study evaluating FCM for IDA treatment in patients with CAG.

In our study, five patients reported adverse reactions (7.35%). Three of these (4.4%) had mild side effects (nausea, dizziness), and the FCM infusion was interrupted. Anyway, the symptoms’ duration was <30 min and did not require further treatment or hospitalisation. Two patients (2.9%) reported mild side effects (nausea, flushing, and skin eruption) not requiring FCM interruption and were quickly treated with steroids and antihistamines. No one reported severe side effects or required hospitalisation.

Common adverse reactions (one patient in ten) reported in various studies vary between 1% and 10% [32].

In a single-centre open-label study on 24 Chinese patients with IDA [33], the incidence of FCM-induced adverse events was 33.3%, comprehending both haematological alterations and symptoms (palpitations, urticaria, nausea, vomiting, abdominal pain, and pyrexia), all of which resolved without sequelae.

In randomised controlled trials on FCM usage in IBD patients’ treatment-emergent adverse events, of any entity, they were reported at 59% and 56.9% [17,18,19]. While in a more recent non-interventional post-marketing study in 101 German centres, only one treatment-emergent adverse event was reported and was a reactivation of disease [22].

Other studies evaluating FCM use in various clinical conditions reported a percentage of FCM hypersensitivity ranging between 0.3% and 0.8% [34,35,36,37,38,39].

Therefore, our finding of 7% of adverse events with FCM interruption in 4% of patients seems to be kept in line with previous results, although the different range of adverse events considered assessed in different clinical settings makes comparison difficult, probably due to the inclusion of haematological alterations and probably different genetic characteristics of the included study population.

One relevant haematological adverse event is hypophosphatemia. This was thought to be transient and asymptomatic [16,37], but it is gaining importance in the last few years due to some studies showing how FCM-related hypophosphatemia could be long-lasting and symptomatic, with an incidence rate between 41% and 70% [38]. In a recent retrospective study on 3 years of experience with FCM, hypophosphatemia was reported in 22.7% of patients and severe hypophosphatemia in 1.1% [39].

Unfortunately, in this study, hypophosphatemia was not evaluated, as discussed later as a limitation. The relatively high number of infusion interruptions in our study must be interpreted given the outpatient setting of the FCM infusion, which requires particular attention to even minimal clinical reactions. No severe reactions were observed in our study, and this might be due to our infusion protocol with a duration of 30 min instead of the indicated 15 min; in fact, rapidity of infusion is known to be associated with a greater probability of moderate-severe reactions [36,40].

Most of the studies evaluating FCM efficacy in IBD patients define response as an increase in Hb levels ≥2 g/dL. This endpoint was achieved both in randomised controlled trials [17,18,19] and in clinical practice studies [20,21,22], mostly using a follow-up time for efficacy evaluation of 12 weeks. In a randomised controlled trial, already at 2 weeks, nearly 5% of patients had an increase in Hb levels ≥2 g/dL.

Stein et al. [22] found a normalization in Hb levels or an increase ≥2 g/dL in Hb levels in 63.3% of patients. Another clinical practice study in IBD patients showed Hb levels normalization in up to 75% [20] of patients at last observation carried forward, with median Hb level ranging from 11.1 g/dL to 11.4 g/dL at baseline in the two study groups and post-treatment values ranging from 13.4 g/dL to 13.2 g/dL. While Kulnigg [18] found an increase from baseline to 12 weeks of 3.7 g/dL of Hb. In a study evaluating 2584 patients with chronic kidney disease randomised between FCM and iron sucrose, the mean Hb increase was 1.13 g/dL [34].

In our study, we found an increase in Hb levels of 2.4 g/dL at 4 weeks and of 3.1 g/dL at 12 weeks, thus showing a good response to FCM treatment in CAG compared to that in IBD patients and superior in the majority of cases. Our data also show a normalization of Hb levels in 78% of patients at 12 weeks, with 67.3% of patients found non-anaemic already at 4 weeks, comparable to the results found by Befrits et al. [20].

Looking at iron storage, our results showed an initial increase of ferritin from 13 ng to 108 ng at 4 weeks, with a decrease to 47 ng/mL at 12 weeks. While transferrin saturation increased from 6.1% to 21.2%, substantially reaching a plateau at 12 weeks. This agrees with what was found in IBD patients concerning ferritin by Stein [22]. In this clinical practice study, the ferritin increase was more pronounced (+51 μg/L) at 4–8 weeks from treatment. In the same study, transferrin saturation increased progressively, differently from what was shown from our data, reaching a final value of 24.2%. The trend shown in our results may be representative of the initial spurt of iron storage, later diminished by iron utilisation in hemopoiesis. This might be due to an insufficient iron dosage administered to our patients. A limitation of the current study is that the dosage of 1000 mg or 700 mg was based exclusively on anaemia severity, not considering other parameters such as body weight as suggested by the Ganzoni formula [41]. A split administration in two sessions might be considered in patients requiring a high dosage to ensure a prolonged effect on iron restoration.

A similar study conducted by Salvadori et al. in patients with inflammatory rheumatic diseases suggested the use of the Ganzoni formula or the body weight for the calculation of FCM dosage to increase the efficacy of treatment [42].

Unfortunately, in the current study, the dosage was not calculated with the Ganzoni formula to simplify the procedures due to logistical needs.

In the current study, anaemia relapse was found in 55.6% of patients after a long period of maintenance (around 2 years). Studies considering the time of maintenance after FCM treatment are few. The FERGI main study [19] evaluated the recurrence of anaemia in IBD patients who received FCM with a maximum follow-up of 8 months. In this study, the recurrence rate at 8 months was 26.7%, which appears to be higher than what was shown in our study if related to the period evaluated. This discrepancy, observed both for efficacy and relapse, may be due to the difference between the two conditions analysed: while CAG is an exclusively malabsorptive disease, IBD may be associated with intestinal bleeding, justifying an earlier recurrence of anaemia. In our series, in fact, just 3% of relapses occurred for bleeding causes not related to CAG. Anyway, these two results are not suitable for direct comparison due to the different diseases evaluated, the different characteristics of the study population, the different periods of analysis, and the different dosages used.

In multivariate analyses, the only factor associated with relapse was the female gender. This result was adjusted for ages > 55 years, so it is assumed to be independent of fertile status and menses. This result is not easy to explain; it might be due to a possible less consistent constitutional iron storage in women or to a gender- or sex-related predisposition (lifestyle, food habits, genetic or hormonal factors) to IDA recurrence. In this context, it might be mentioned that autoimmune gastritis is much more frequent in women, in whom it manifests mainly with IDA, while in men it is more often characterised by the presence of pernicious anaemia [43].

We are aware of some limitations of the current study, this is, in fact, a monocentric retrospective study, a considerable point of strength given the homogeneity of patients’ management and data acquisition. Moreover, the menopausal status was not available for all patients but just a subset of them. Phosphate levels were not evaluated, neither at baseline nor after FCM infusion, allowing the assessment of post-FCM hypophosphatemia. Finally, our study population includes cases of concomitant IDA and pernicious anaemia, possibly representing a bias; however, in multivariate analyses, the role of cobalamin deficiency in determining IDA relapse was excluded.

In conclusion, in this retrospective monocentric experience, FCM appeared to be well tolerated and effective in treating IDA in patients with CAG, ensuring recovery from IDA in 80% of treated patients at 3 months from infusion with good maintenance free of anaemia, up to 2 years in half of patients. Women result in relapse more frequently, apparently irrespective of age > 55 years. Further studies are welcome to understand the reasons behind this relationship between the female gender and IDA relapse.

## Figures and Tables

**Table 1:** Haemoglobin, ferritin, and transferrin saturation trends and values at T0, T4, and T12.

	T0(Tf)	T4	*p*-Value T0–T4	T12	*p*-Value T4–T12	*p*-Value T0–T12
Hb (g/dL ± SD)	10.23 ± 1.7	12.62 ± 1.42	<0.0001	13.28 ± 1.3	0.0547	<0.0001
RDW (% ± SD)	17.6 ± 6	21.9 ± 10.9	0.0542	16.63 ± 4.8	0.0013	0.0229
Ferritin (ng/mL ± SD)	12.9 ± 29.3	108.2 ± 106.4	<0.0001	46.95 ± 74.9	<0.0001	0.3151
Transferrin saturation (% ± SD)	6.12 ± 3.9	21.25 ± 7.85	=0.0001	18.67 ± 9.95	0.7021	<0.001

**Table 2:** Principal characteristics of patients with relapse and patients without relapse.

	Relapse	Non-Relapse	*p*-Value
Female	50.8%	27.7%	0.0042
Age > 55 years old	27.7%	16.9%	0.3343
NSAIDs	10.9%	14.1%	0.2482
Cobalamin supplementation	40.3%	27.4%	0.179
Severe atrophy	16.9%	21.5%	0.1475
Severe anaemia	3.1%	3.1%	0.8244

## Data Availability

The data presented in this study are available on request from the corresponding author. The data are not publicly available due to privacy reasons.
